# Genetic analysis of Behçet’s disease using whole-exome sequencing and bioinformatics analysis in Korean patients

**DOI:** 10.3389/fgene.2026.1787087

**Published:** 2026-08-14

**Authors:** Enkhjargal Bayarsaikhan, Hyunsoo Ahn, Sanguk Kim, Yong-Beom Park, Jae Hoon Lee

**Affiliations:** 1 Department of Prosthodontics, College of Dentistry, Yonsei University, Seoul, Republic of Korea; 2 Graduate School of Artificial Intelligence, Pohang University of Science and Technology, Pohang, Republic of Korea; 3 Department of Life Sciences, Pohang University of Science and Technology, Pohang, Republic of Korea; 4 Department of Internal Medicine, College of Medicine, Yonsei University, Seoul, Republic of Korea

**Keywords:** Behçet’s disease, bioinformatics, exome sequencing, functional enrichment analysis, protein-protein interaction network, susceptibility genes

## Abstract

**Background:**

Behçet’s disease (BD) is a rare autoimmune or autoinflammatory disorder characterized by various systemic manifestations, including mucocutaneous, ocular, and musculoskeletal symptoms. The etiology of BD involves a complex interplay between genetic predisposition and environmental factors.

**Methods:**

In this study, we conducted whole-exome sequencing of 20 Korean patients with BD to identify putative genetic markers and assess their association with the disease.

**Results:**

We identified six genes, namely *TTN, FOXO3, OR4C5, GXYLT1, ERN1*, and *SIPA1L3*, harboring variants with high odds ratios and significant associations with BD. Network-based analysis revealed 48 candidate disease genes that interacted with the identified target genes. These genes were enriched in the interleukin and cytokine signaling pathways, suggesting their potential involvement in BD pathogenesis.

**Conclusion:**

Our findings shed light on the genetic basis of BD and provide insights into its molecular mechanisms, paving the way for further research and targeted therapeutic interventions.

## 1 Introduction

Behçet’s disease (BD) is a rare, chronic, complex condition characterized by various manifestations, including mucocutaneous, ocular, and musculoskeletal symptoms. It is often accompanied by recurrent oral aphthous ulcers with occasional systemic manifestations, including vascular, gastrointestinal, respiratory, and central nervous system symptoms (Barnes and Yazici, 1999; Sakane et al., 1999; Kurokawa et al., 2004). BD is considered an autoimmune and autoinflammatory disorder, the latter refers to inherited disorders characterized by recurrent episodes of innate immune system inflammation, often involving neutrophils, without clear triggers (Direskeneli, 2006; Takeuchi et al., 2015).

BD typically presents between the ages of 20 and 40 years and affects both men and women equally (Leccese and Alpsoy, 2019). However, its diagnosis is challenging because of the absence of a universally accepted laboratory test as it is typically based on clinical symptoms and signs. To aid in diagnosis, classification criteria, such as the International Study Group (ISG) (International Study Group for Behçet’s Disease, 1990) and the International Criteria for Behçet’s Disease (ICBD) (International Team for the Revision of the International Criteria for Behcet’s Disease, 2006) are commonly used and widely recognized internationally (Davatchi, 2012). BD is most prevalent along the Silk Road, particularly in Turkey and Iran, where rates reach 421 per 100,000 people, although its global incidence is rising, notably in South Korea (Verity et al., 1999; Cho et al., 2012).

BD is believed to be triggered by environmental factors, such as viral and bacterial infections, in individuals with a genetic predisposition (Mendes et al., 2009). Although the exact cause is not fully understood, previous studies have reported a higher prevalence of tonsillitis and dental caries in patients with BD (Aoki and Ono, 1972), suggesting possible lymphocyte sensitization to streptococcal antigens (Martin et al., 1979). Herpes simplex virus has also been detected in the saliva and ulcerative lesions of patients with BD using polymerase chain reaction (PCR) (Lee et al., 1996).

The major histocompatibility complex or human leukocyte antigen (HLA) region is strongly associated with autoimmune diseases, and *HLA-B51* has been identified as a genetic factor in BD (Verity et al., 1999; de Menthon et al., 2009). However, its incomplete penetrance necessitates the investigation of other susceptibility genes, including the identified risk factors *HLA-B15*, *HLA-B26*, *HLA-B27*, and *HLA-B57* (Takeuchi et al., 2015). Genome-wide association studies (GWAS) have identified non-HLA genes, including *IL10*, *IL23R*, *IL12RB2*, *STAT4*, *CCR1*, *ERAP1*, and *KLRC4-KLRK1*, that are associated with susceptibility beyond the HLA region (Mizuki et al., 2010; Remmers et al., 2010; Hou et al., 2012; Kirino et al., 2013). However, ethnic differences exist because certain genes that are significant in the Korean population have not been shown to be significant in other populations (Lee et al., 2013; Ortiz-Fernández et al., 2014).

Despite these findings, the genetic aspects of BD have not yet been fully elucidated, particularly in patients with similar symptoms and unclear pathogenesis. Next-generation sequencing, such as whole-exome sequencing (WES), offers an alternative approach for investigating the relationship between BD symptoms and genetic biomarkers. By targeting the protein-coding regions (exons) of the genome, WES can be used to investigate various genetic variants, including homozygous, heterozygous, *de novo*, germline, and rare novel coding variants, specifically focusing on non-synonymous mutations that can affect protein function. This approach overcomes the limitations of previous GWAS by detecting both structural and rare variants. Furthermore, the application of protein functional network and gene set enrichment analyses to WES data allows for the classification of variants into functionally similar gene clusters (Johar et al., 2015).

This study aimed to identify genetic biomarkers associated with BD and elucidate the functional relationships between these genetic associations and the pathogenesis of the disease. WES was performed on individuals with BD to identify potential variants at specific loci, which were then matched with the corresponding BD phenotypic symptoms in the Korean population. Subsequently, bioinformatics tools such as gene set enrichment analysis, followed by statistical analysis, were used to determine the pathogenesis of BD.

## 2 Materials and methods

### 2.1 Participants

We recruited 20 Korean patients who had been diagnosed with BD at the Rheumatology Clinic of Severance Hospital. All patients fulfilled the ICBD and were unrelated to each other. A medical examination form was used to collect clinical signs, symptoms, complications, and laboratory results. Patients were interviewed to determine whether they experienced symptoms, such as oral aphthosis, ocular lesions, skin lesions, genital aphthosis, vascular manifestations, neurological manifestations, or positive pathergy test results. Patients who had been diagnosed with other autoimmune diseases were excluded.

### 2.2 Ethics statement

The Institutional Review Board of Yonsei University Dental Hospital approved all research involving human participants and data (IRB No. 2-2019-0040), and all clinical investigations were performed in accordance with the Declaration of Helsinki. Written informed consent was obtained from all participants prior to their enrollment.

### 2.3 Sample collection

Saliva was collected from each patient using an Oragene DNA Self-Collection Kit (DNA Genotek Inc. Ottawa, Canada) according to the manufacturer’s instructions. Each participant was asked to provide 2 mL of saliva in an Oragene DNA Self-Collection Kit tube containing a DNA-preserving solution. The lid was then closed to mix the saliva with the storage liquid, enabling the saliva samples to remain stable at room temperature. DNA Link Inc. (Seoul, South Korea) performed genomic DNA collection, DNA extraction, and further analysis.

### 2.4 Control group

One hundred healthy Koreans from the 3,703 individuals in the Ansan-Ansung cohort were selected as a randomized subsample using exome sequencing data provided by the Korea BioBank, Center for Genome Science, National Institute of Health, and Korea Centers for Disease Control and Prevention. To ensure a healthy comparison, individuals with a history of malignancy, chronic inflammatory diseases, or known autoimmune disorders were excluded.

### 2.5 WES

A SureSelect Human All Exon 50 MB Kit (Agilent, Santa Clara, CA, United States) was used. Genomic DNA was extracted, and its quality was confirmed using 1% agarose gel electrophoresis and a PicoGreen® dsDNA Assay. The DNA was undamaged, with an OD260/280 ratio of 1.8–2. To prepare SureSelect sequencing libraries, 3 μg of genomic DNA was fragmented to a median size of 150 bp using a Covaris-S2 instrument. Fragmentation efficiency was evaluated using capillary electrophoresis on DNA 1000 chips. Sequencing adapters were ligated to the DNA fragments, which were then amplified using PCR. The quality of the PCR products was evaluated using capillary electrophoresis. The hybridization buffer was prepared using SureSelect hyb #1, #2, #3, and #4 reagents. The amplified DNA fragments were concentrated to aliquots of 500 ng in 3.4 µL with SureSelect block #1, #2, and #3 reagents mixed. The DNA blocker mix and hybridization buffer were incubated, followed by the addition of the RNase block to the SureSelect oligo capture library, which was then incubated. Subsequently, the hybridization buffer containing the DNA blocker mix was added to the capture library, and the mixture was incubated. The hybridization mixture was added to resuspended beads coated with Dynal MyOne Streptavidin T1 and incubated. The beads were washed, and the captured DNA was eluted using SureSelect Elution Buffer, followed by the addition of SureSelect Neutralization Buffer. The reaction products were purified using AMPure XP beads. The captured library was then amplified to add index tags using Herculase II Fusion DNA Polymerase. The quality of the amplified libraries was verified using capillary electrophoresis. Six libraries were combined, index-tagged in equimolar amounts, and then pooled. Cluster generation occurred in the flow cell of the cBot automated cluster generation system, and the flow cell was loaded onto an Illumina NovaSeq 6000 sequencing system (Illumina) and sequenced with a 2 × 100 bp read length. Data were processed using GATK v3.5 (Van der Auwera et al., 2013) for variant calling, with sequence reads aligned to the human reference genome (hg19).

### 2.6 Analysis

#### 2.6.1 Identification of candidate biomarkers and statistical assessment

Statistical analyses were performed to identify candidate variants associated with BD. The association between variants and BD was calculated using Fisher’s exact test, as implemented in PLINK v1.07 (Purcell et al., 2007), followed by allelic testing for association testing. Variants with call rates < 0.85 were discarded for quality control. Furthermore, variants with a minor allele frequency (MAF) > 0.01 were excluded using the Genome Aggregation Database (gnomAD) v2.1.1 dataset (Karczewski et al., 2020) to retain only the rare variants observed exclusively in patients with BD. A variant that was strongly enriched (odds ratio [OR] > 1, *p* < 0.001) in patients with BD was considered to be significant. To filter the BD candidate variants that were potentially pathogenic, we validated the functional impact of variants on protein-coding regions and retained variants with HIGH/MODERATE impact using SnpEff (Cingolani et al., 2012), derived from pre-calculated scores in the dbNSFP database (Liu et al., 2020).

Fisher’s exact test was used for between-group comparisons to assess the statistical significance of each candidate variant across patient subgroups. The OR with a 95% confidence interval (CI) was calculated for seven phenotypic subgroups distinguished by seven types of patient symptoms: oral aphthosis, ocular lesions, skin lesions, genital aphthosis, vascular manifestations, neurological manifestations, and a positive pathergy test. All statistical analyses were performed using SciPy v1.1.0 (Virtanen et al., 2020) and Pandas v0.23.1 (McKinney, 2010) using Python v2.7.13.

#### 2.6.2 Network-based functional enrichment analysis

**FIGURE 1:**
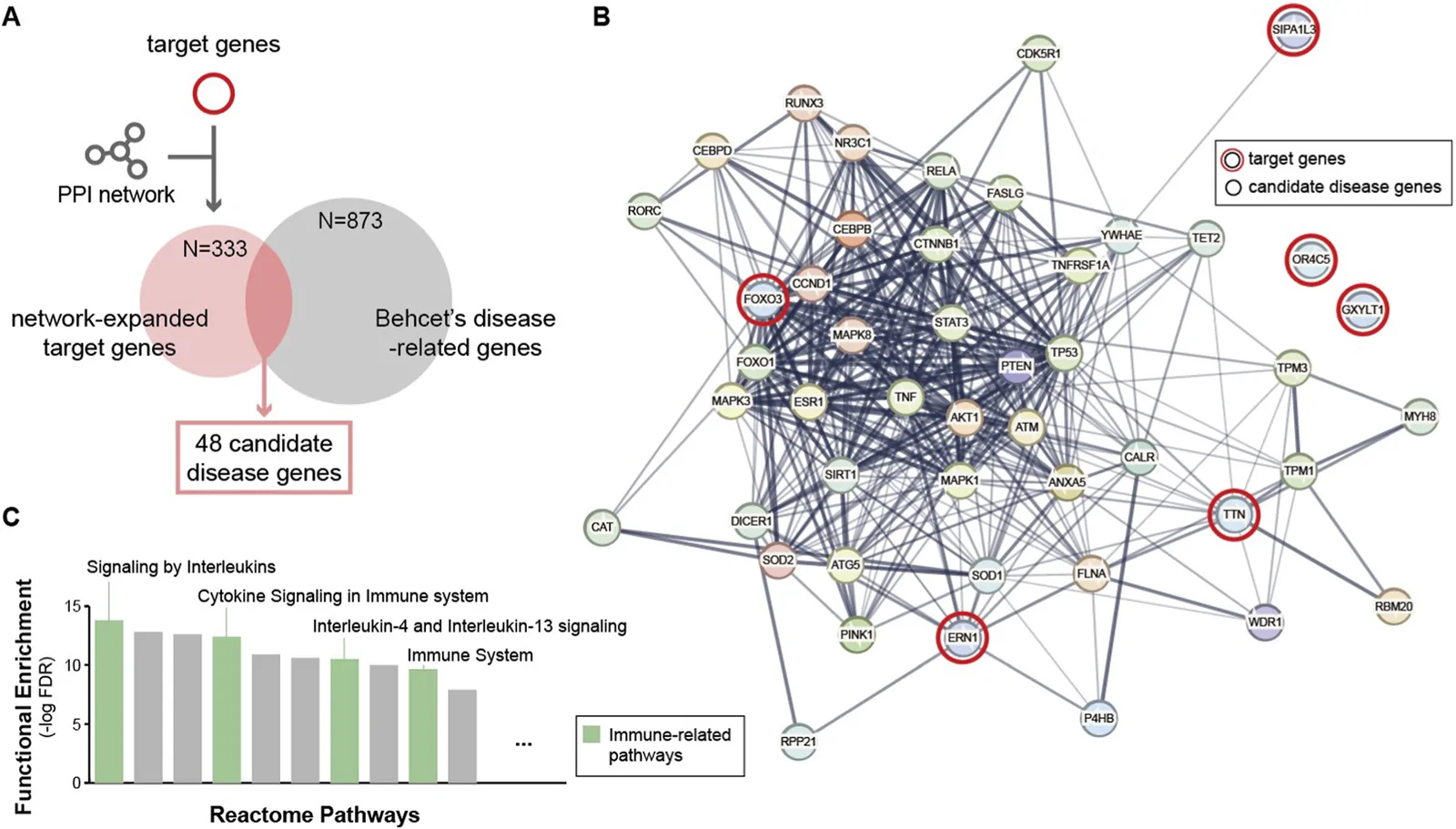
Network-based candidate disease genes of BD. **(A)** Scheme for extracting candidate disease genes. Intersecting genes between network-expanded target genes and BD-related genes were selected. **(B)** Protein-protein interaction network of the candidate disease genes. The target genes are highlighted in red circles. **(C)** Functional enrichment analysis of the candidate disease genes. The top 10 most significantly enriched terms of the Reactome pathway are visualized. Immune-related pathways are highlighted in green.

Genes in which the 24 filtered variants were located specifically in their protein-coding regions were considered target genes. Genes that interacted with the target genes and were concurrently reported as BD-related genes were identified as potential disease genes. Target genes were expanded with the first neighbors in the protein-protein interaction network as network-expanded target genes. The network was constructed using STRING (Szklarczyk et al., 2022) with a high-confidence combined score cutoff of 0.7, incorporating all available interaction evidence. Genes related to BD were identified based on their known associations with BD, using the whole associated-target data for the term “Behçet’s syndrome” in the Open Targets database (Ochoa et al., 2023) and “Behçet Syndrome” in the DisGeNET database (Piñero et al., 2020). The genes directly intersecting the network-expanded target genes and BD-related genes were considered candidate disease genes in the cohort (Figure 1A). The g:Profiler (Kolberg et al., 2023) was used to perform Reactome pathway (Griss et al., 2020) term enrichment for the candidate disease genes, using all annotated genes of humans in the Ensembl (Harrison et al., 2024) database. The terms significantly enriched in the candidate disease genes were selected with a false discovery rate (FDR) < 0.05 using the g:SCS algorithm (Kolberg et al., 2023).

To assess whether the candidate disease genes reflect disease-specific associations rather than generic network hub effects, degree centrality and PageRank were computed for all candidate disease genes within the STRING network using NetworkX v3.6.1 (Hagberg et al., 2008). Degree centrality was defined as the proportion of nodes to which a given node is directly connected. PageRank was computed with a damping factor of 0.85 and edge weights corresponding to normalized STRING combined scores. All 38 candidate disease genes present in the network were ranked against the full network of 15,134 nodes.

## 3 Results

### 3.1 Characteristics of the study cohort

The baseline characteristics of the patient and control groups are presented in Table 1. While the two groups were statistically similar in age (mean 56.15 ± 10.17 vs. 52.3 ± 8.1 years; *p* = 0.068), a significant difference was observed in sex distribution (85.0% female in patients vs. 52.0% in controls; *p* = 0.006). To account for these differences and minimize potential bias, age and sex were included as covariates in all subsequent genetic association analyses. Clinical and laboratory features of the 20 patients were presented in Table 2.

**TABLE 1:** Baseline characteristics of participants.

Characteristic	Patients (n = 20)	Controls (n = 100)	*p*-value
Age (years, mean ± SD)	56.15 ± 10.17	52.3 ± 8.1	0.068
Sex, n (%)	​	​	0.006
Female	17 (85%)	52 (52%)	​
Male	3 (15%)	48 (48%)	​

**TABLE 2:** Laboratory and clinical features of the 20 patients.

Variable	Patients (%)
Age of the disease onset (years, mean ± SD)	36.15 ± 11.77
Laboratory feature	
Positive pathergy test	2/20 (10)
Clinical manifestations	
Oral aphthosis	20/20 (100)
Ocular lesions	14/20 (70)
Skin lesions	16/20 (80)
Genital aphthosis	18/20 (90)
Vascular manifestations	4/20 (20)
Neurological manifestations	2/20 (10)

### 3.2 Exome-wide association analysis

This study aimed to identify risk variants associated with BD through exome-wide association analysis. After applying sequential filtering criteria–excluding variants with call rate <0.85, retaining only rare variants (MAF < 0.01 in gnomAD), and selecting those with HIGH/MODERATE functional impact (see Methods)—variants located in six genes (*TTN*, *FOXO3*, *OR4C5*, *GXYLT1*, *ERN1*, and *SIPA1L3*) met the threshold of p < 5 × 10^−5^ and OR > 1 (Table 3). The highest OR was observed for the *ERN1* single nucleotide polymorphism (SNP) rs150831277, with an OR of 199 (95% CI). The lowest association p-values (p = 3.01 × 10^−11^) were found for the *GXYLT1* SNP rs181558534, followed by *OR4C5* SNP rs10769651 (p = 5.32 × 10^−10^), *FOXO3* SNP rs34133353 (p = 8.03 × 10^−6^), and *TTN* SNP rs75615667 (p = 3.22 × 10^−5^). Notably, *OR4C5*, *ERN1*, and *SIPA1L3* genes have not been previously associated with BD.

**TABLE 3:** SNPs in the exome-wide association analysis.

Gene	SNP	CHR	POS	REF	ALT	MAF		Discovery		AA change
						Case	Control	OR (95% CI)	*P*	
*TTN*	rs72650066	2	179515483	G	C	0.333	0.063	7.417	3.22E-05	p.Pro13235Arg
*FOXO3*	rs34133353	6	108985176	T	TG	0.194	0.005	48.03	8.03E-06	p.Leu382fs
*OR4C5*	rs10769651	11	48387668	C	G	0.425	0.034	21.19	5.32E-10	p.Gly117Ala
*GXYLT1*	rs181558534	12	42538352	C	A	0.421	0.068	58.04	3.01E-11	p.Gly33*
*ERN1*	rs150831277	17	62121452	T	C	0.5	0.005	199	8.64E-04	p.Thr944Ala
*SIPA1L3*	rs371571630	19	38572367	G	GGCCACC	0.125	0.005	28.43	5.44E-03	p.Ala63_Thr64dup

SNP, single nucleotide polymorphism; OR, odds ratio; CHR, chromosome; POS, base pair position; REF, reference allele; ALT, alternative allele; MAF, minor allele frequency; CI, confidence interval; P, p-value of association analysis; AA, amino acid.

### 3.3 Genes identified in the subgroup of patients

Because the allelic heterogeneity and homogeneity of these nine variants were significant for BD but did not contribute to all tests, subgroup-specific associations and frequencies may vary by subgroup. Supplementary Tables S1–S7 summarize the genes in the BD symptom subgroups. With the highest OR of 4.18, *GXYLT1* expression was more prevalent in patients with genital aphthosis than in those without symptoms (Supplementary Table S4).

### 3.4 Network-based candidate disease gene analysis

Network analysis revealed that mutations in the cohort affected the interleukin signaling pathway. Because proteins function by interacting with several other proteins, variants with a high impact, such as amino acid substitutions, interfere with the functions of the proteins that interact with them. Using network-based analysis, we identified 48 candidate disease genes related to BD that were affected by the target genes in which the mutations found in the cohort were located (Figure 1A). The target genes *TTN*, *FOXO3*, *ERN1*, and *SIPA1L3* interacted with genes reported to be BD-related, whereas *GXYLT1* and *OR4C5* did not (Figure 1B). Candidate disease-related genes were significantly enriched for functions related to “Signaling by Interleukins,” “Cytokine Signaling in the Immune System,” and “Interleukin-4 and Interleukin-13 Signaling” in the Reactome pathways (Figure 1C). This result indicates that the candidate variants observed in the cohort affected the interleukin signaling pathways, which might extend to BD.

To evaluate whether candidate disease genes were selected for disease-specific associations rather than network hub bias, degree centrality and PageRank were computed for all candidate disease genes in the STRING network. While the median PageRank percentile across all candidate genes was 90.3%, the distribution was notably heterogeneous. A subset of genes, including *DAPK3* (30.0th percentile), *TET2* (41.1st percentile), *NR2F2* (47.3rd percentile), and *WDR1* (47.2nd percentile), exhibited low network centrality, indicating that their selection was driven by BD-specific association evidence from Open Targets and DisGeNET rather than by high connectivity alone. This heterogeneous centrality profile supports the disease-specificity of the candidate gene set and argues against systematic hub bias in our network-based approach (Supplementary Figures S1 and S2).

## 4 Discussion

We performed a case-control genetic association analysis using WES data from 20 patients with BD and 100 healthy controls. This is one of the first WES-based exploratory studies on rare variants in Korean patients with BD to determine their genetic susceptibility. Notably, we identified six susceptibility loci for BD, with *TTN*, *FOXO3*, *OR4C5*, *GXYLT1*, *ERN1*, and *SIPA1L3* being significant contributors. What is particularly striking is that the *OR4C5*, *ERN1*, and *SIPA1L3* genes have not been previously implicated in BD, signifying a pivotal advancement in our understanding of the genetic architecture of this complex disorder.

The *TTN* gene encodes the autoantigen titin, which is broadly implicated in immune dysregulation across multiple autoimmune conditions, including myasthenia gravis and autoimmune rippling muscle disease (Somnier et al., 1999; Zelinka et al., 2011). Our identification of *TTN* as a risk-associated gene aligns with emerging genomic evidence, such as the whole-exome sequencing of European cohorts (Ognenovski et al., 2016). However, while Ognenovski et al. identified rare variants in *TTN*, they ultimately focused their conclusions on *NOD2* and *MEFV*—two genes that did not meet our significance thresholds. Several critical factors likely explain these disparate profiles. First, ethnic divergence is highly pronounced in BD genetics; our cohort consists entirely of Korean (East Asian) populations, whereas the previous study examined European populations. Second, diagnostic thresholds varied: the European study utilized the ISG criteria, which heavily mandates oral ulceration, whereas our study employed the ICBD, which exhibits higher sensitivity by assigning weighted scores to vascular and neurological manifestations. Finally, baseline demographics differed starkly; our cohort had a strong female predominance (85%), whereas western cohorts often demonstrate more balanced or male-skewed ratios. These distinct clinical and demographic baselines underscore how cohort composition influences the detection of rare variants.

In this study, *FOXO3* exhibited a statistically significant but imprecise OR due to the small sample size. Our investigation into the involvement of *FOXO3* in BD revealed its pivotal role in modulating inflammatory pathways and immune responses. The regulatory proficiency of *FOXO3* in inflammation, as evidenced by its effect on diseases such as rheumatoid arthritis, underscores its significance as a potential therapeutic target for autoimmune conditions (Xu et al., 2021; Wang et al., 2020; Kadiri et al., 2021). Furthermore, *FOXO3* has been increasingly recognized as a critical immune regulator. A recent study identified that *FOXO3* was linked to BD associated-atherosclerosis using machine learning, suggesting that this gene may serve as a biomarker and therapeutic target for BD-induced atherosclerosis pathogenesis (Li et al., 2025). *FOXO3* plays a crucial role in modulating Th17-mediated autoimmunity (Chen et al., 2022). While the genetic association in a Korean BD cohort is a novel observation of this study, the functional relevance of *FOXO3* is supported by its established role in Th17 modulation. However, given that our identified variants were not perfectly correlated with specific clinical manifestations—and acknowledging that our subgroup numbers are very small—extreme caution must be exercised. It is therefore premature to conclude that these specific *FOXO3* variants can independently serve as diagnostic biomarkers or definitive therapeutic targets for BD. Instead, they should be viewed as preliminary candidates that require robust evaluation in much larger, statistically powered cohorts.

In our study, the *OR4C5* (Olfactory Receptor Family 4 Subfamily C Member 5) gene showed a highly significant p-value. However, no interaction was observed between BD-related genes and *OR4C5* in this study. Interestingly, olfactory dysfunction has previously been associated with BD (Veyseller et al., 2014). Their findings suggest that neurological manifestations associated with BD may play a more prominent role in olfactory dysfunction than mucosal manifestations. Moreover, olfactory function is impaired in both neurological manifestations of BD and neuro-BD (Gencer Culha and Dogan, 2025). Another study observed reduced olfactory identification in patients with BD (Akyol et al., 2016).

The relationship between *GXYLT1* and autoimmune diseases, including Cronkhite-Canada syndrome (Cronkhite and Canada, 1955; Daniel et al., 1982; Liu et al., 2022), elucidates its role in chronic autoimmune inflammation and colorectal carcinogenesis. In our study, *GXYLT1* expression was significantly higher in patients with genital aphthosis (Supplementary Table S4), suggesting a potential link between this gene and specific disease phenotypes. In a previous study, *GXYLT1* was shown to be involved in the glycosylation-related inflammatory pathways. *GXYLT1*, a glycosylation pathway gene, might also serve as a diagnostic marker for subtype identification (Zhan et al., 2023). *GXYLT1*, whose biological link to BD remains tentative, requires further functional validation.

Although *ERN1* may not be directly linked to autoimmune diseases, previous studies have revealed its potential as a therapeutic target. The use of small interfering RNA drugs targeting *ERN1* has shown potential for treating autoimmune inflammatory disorders, underscoring the translational implications of our findings (Feng et al., 2021).

Our exploration of the indirect association between *SIPA1L3* and autoimmune diseases through gut microbiome interactions provides intriguing insights into the intricate interplay between host genetics and environmental factors. By elucidating the role of *SIPA1L3* variants in influencing gut microbiota composition and susceptibility to diseases, such as immunoglobulin A nephropathy (He et al., 2021), our study highlights the multifaceted nature of autoimmune diseases and the need for a comprehensive understanding of their etiology. Previous studies have found that *SIPA1L3* is critical for eye development (Greenlees et al., 2015), and that mutations in this gene are associated with congenital cataracts (Evers et al., 2015). A meta-analysis of Chinese and Japanese cohorts revealed novel risk loci, including *SIPA1-FIBP-FOSL1*, in BD-related uveitis, with a significant association (Su et al., 2022). Given that *SIPA1L3* is essential for ocular development and has been linked to congenital cataracts, its identification as a susceptibility locus in our cohort warrants further investigation into its specific role in BD-related uveitis.

Finally, the most significantly enriched terms related to immunity in our network analysis involved interleukin and cytokine signaling pathways, specifically pointing to Interleukin-4 (IL-4) and Interleukin-13 (IL-13) signaling. Because BD is classically characterized as a Th1 and Th17-driven inflammatory disorder (Lopalco et al., 2017; Hagberg et al., 2008), the enrichment of these typical Th2 pathways may initially appear counterintuitive, likely reflecting a network artifact stemming from our modest sample size. However, a more detailed biological link exists through shared intracellular machinery. IL-4 and IL-13 signal transduction pathways share critical upstream receptor chains (such as IL-4Rα) and downstream regulatory cascades, including the JAK/STAT pathways, which directly overlap with the signaling architecture of pro-inflammatory cytokines (Jiang et al., 2000; Bao et al., 2013). Furthermore, IL-4 functions as a primary anti-inflammatory cytokine; previous studies have noted that dysregulated IL-4 levels or IL-4 receptor polymorphisms can impair the host’s ability to suppress aberrant Th1/Th17 activation in BD (Shahram et al., 2011; Inanir et al., 2013). Therefore, the enrichment observed in our network analysis likely captures downstream disruptions in shared immune-regulatory networks rather than an active Th2-mediated disease process, a nuance that warrants precise functional validation in larger cohorts.

In summary, this study expands the genetic landscape of autoimmune and autoinflammatory diseases and highlights the complex interplay between genetic predisposition, immune dysregulation, and environmental factors that drive BD susceptibility. These insights provide a foundation for the development of novel therapeutic interventions and personalized treatment strategies. However, these findings should be interpreted within the context of several limitations. First, the modest sample size may have constrained the statistical power to detect low-frequency variants. Additionally, the sex imbalance—with a predominantly female patient cohort—may limit the generalizability of our findings to males. While we adjusted for sex in our statistical models, future studies with larger, sex-matched cohorts are necessary for validation. Finally, although the current study lacked functional characterization and Sanger sequencing of WES-derived variants, it establishes a critical framework for future large-scale prospective validation studies.

## 5 Conclusion

Our genetic analysis identified a significant prevalence of variants in six key genes, namely, *TTN*, *FOXO3*, *OR4C5*, *GXYLT1*, *ERN1*, and *SIPA1L3*, in patients with BD. These genes are integral to essential biological pathways, including immune modulation, inflammatory signaling, and cell cycle regulation. Therefore, elucidation of the precise mechanistic roles of these candidates in BD pathogenesis is imperative. These efforts are vital for developing robust diagnostic biomarkers and targeted therapeutic approaches for complex multisystem disorders.

## Data Availability

The original contributions presented in the study are publicly available. This data can be found in the NCBI repository with the accession number PRJNA1481407.
