# Supplementary material for: Genetic analysis of Behçet’s disease using whole-exome sequencing and bioinformatics analysis in Korean patients
Source: Front Genet. 2026 Aug 14;17:1787087. doi: 10.3389/fgene.2026.1787087 (PMC13521551; doi:10.3389/fgene.2026.1787087)
Supplement: Supplementary file 1 [file DataSheet1.docx]

# 1 Supplementary Figures

**Supplementary Figure 1.** Scatter plot of Degree and PageRank percentile for candidate disease genes. High/Mid/Low-centrality genes are classified (Blue, gray, and orange,respectively). Low-centrality genes are annotated.

**Supplementary Figure 2.** Histogram of PageRank percentile distribution. High/Mid/Low-centrality genes are classified (Blue, gray, and orange, respectively). Thresholds are presented with dashed lines.

# 2 Supplementary Tables

**Supplementary Table 1.** Summary of genes in the oral aphthosis subgroups. Note: 'Normal' refers to the patients diagnosed with BD who do not exhibit oral aphthosis.

| Gene | Oral aphthosis  (total = 20) | Normal  (total = 0) | OR | 95% CI | P |
| --- | --- | --- | --- | --- | --- |
| *TTN* | 10/18 (56%) | 0/0 (-%) | not estimable | N/A | N/A |
| *FOXO3* | 7/18 (39%) | 0/0 (-%) | not estimable | N/A | N/A |
| *OR4C5* | 17/20 (85%) | 0/0 (-%) | not estimable | N/A | N/A |
| *GXYLT1* | 15/19 (79%) | 0/0 (-%) | not estimable | N/A | N/A |
| *ERN1* | 2/2 (100%) | 0/0 (-%) | not estimable | N/A | N/A |
| *SIPA1L3* | 5/10 (25%) | 0/0 (-%) | not estimable | N/A | N/A |

OR, odds ratio; CI, confidence interval; P, p-value of association analysis; Inf, infinite (due to zero counts in one or more cells, reflecting the mathematical boundary conditions of Fisher's exact test); N/A, not applicable (due to absence of comparator group).

**Supplementary Table 2.** Summary of genes in the ocular lesion subgroups. Note: 'Normal' refers to the patients diagnosed with BD who do not exhibit ocular lesions.

| Gene | Ocular lesions  (total = 14) | Normal  (total = 6) | OR | 95% CI | P |
| --- | --- | --- | --- | --- | --- |
| *TTN* | 7/13 (54%) | 3/5 (60%) | 0.79 | 0.0-9.6 | 1 |
| *FOXO3* | 4/13 (31%) | 3/5 (60%) | 0.32 | 0.0-4.0 | 0.33 |
| *OR4C5* | 11/14 (79%) | 6/6 (100%) | 0.0 | 0.0-5.8 | 0.52 |
| *GXYLT1* | 10/13 (77%) | 5/6 (83%) | 0.68 | 0.0-11.4 | 1 |
| *ERN1* | 1/1 (100%) | 1/1 (100%) | 1.0 | 0.0-155.9 | 1 |
| *SIPA1L3* | 2/14 (14%) | 3/6 (50%) | 0.19 | 0.0-2.4 | 0.13 |

OR, odds ratio; CI, confidence interval; P, p-value of association analysis; Inf, infinite (due to zero counts in one or more cells, reflecting the mathematical boundary conditions of Fisher's exact test).

**Supplementary Table 3.** Summary of genes in the skin lesion subgroups. Note: 'Normal' refers to the patients diagnosed with BD who do not exhibit skin lesions.

| Gene | Skin lesions  (total = 16) | Normal  (total = 4) | OR | 95% CI | P |
| --- | --- | --- | --- | --- | --- |
| *TTN* | 6/14 (43%) | 4/4 (100%) | 0.0 | 0.0-1.7 | 0.092 |
| *FOXO3* | 4/14 (29%) | 3/4 (75%) | 0.15 | 0.0-2.5 | 0.25 |
| *OR4C5* | 14/16 (88%) | 3/4 (75%) | 2.2 | 0.0-58.1 | 0.51 |
| *GXYLT1* | 11/15 (73%) | 4/4 (100%) | 0.0 | 0.0-6.3 | 0.53 |
| *ERN1* | 2/2 (100%) | 0/0 (-%) | not estimable | N/A | N/A |
| *SIPA1L3* | 3/16 (19%) | 0/2 (0%) | Inf | 0.0-Inf | 1 |

OR, odds ratio; CI, confidence interval; P, p-value of association analysis; Inf, infinite (due to zero counts in one or more cells, reflecting the mathematical boundary conditions of Fisher's exact test); N/A, not applicable (due to absence of comparator group).

**Supplementary Table 4.** Summary of genes in the genital aphthosis subgroups. Note: 'Normal' refers to the patients diagnosed with BD who do not exhibit genital aphthosis.

| Gene | Genital aphthosis  (total = 18) | Normal  (total = 2) | OR | 95% CI | P |
| --- | --- | --- | --- | --- | --- |
| *TTN* | 9/16 (56%) | 1/2 (50%) | 1.27 | 0.0-111.9 | 1 |
| *FOXO3* | 7/16 (44%) | 0/2 (0%) | Inf | 0.1-Inf | 0.50 |
| *OR4C5* | 15/18 (83%) | 2/2 (100%) | 0.0 | 0.0-34.4 | 1 |
| *GXYLT1* | 14/17 (82%) | 1/2 (50%) | 4.18 | 0.0-390.2 | 0.39 |
| *ERN1* | 2/2 (100%) | 0/0 (-%) | not estimable | N/A | N/A |
| *SIPA1L3* | 5/18 (28%) | 0/2 (0%) | Inf | 0.0-Inf | 1 |

OR, odds ratio; CI, confidence interval; P, p-value of association analysis; Inf, infinite (due to zero counts in one or more cells, reflecting the mathematical boundary conditions of Fisher's exact test); N/A, not applicable (due to absence of comparator group).

**Supplementary Table 5.** Summary of genes in the vascular manifestation subgroups. Note: 'Normal' refers to the patients diagnosed with BD who do not exhibit vascular manifestation.

| Gene | Vascular manifestation  (total = 4) | Normal  (total = 16) | OR | 95% CI | P |
| --- | --- | --- | --- | --- | --- |
| *TTN* | 1/3 (33%) | 9/15 (60%) | 0.35 | 0.0-8.3 | 0.56 |
| *FOXO3* | 1/3 (33%) | 6/15 (40%) | 0.76 | 0.0-17.9 | 1 |
| *OR4C5* | 4/4 (100%) | 13/16 (81%) | Inf | 0.1-Inf | 1 |
| *GXYLT1* | 1/4 (25%) | 14/15 (93%) | 0.036 | 0.0-0.8 | 0.016 * |
| *ERN1* | 1/1 (100%) | 1/1 (100%) | not estimable | 0.0-Inf | 1 |
| *SIPA1L3* | 1/4 (25%) | 4/16 (25%) | 1.0 | 0.0-17.6 | 1 |

OR, odds ratio; CI, confidence interval; P, p-value of association analysis; Inf, infinite (due to zero counts in one or more cells, reflecting the mathematical boundary conditions of Fisher's exact test).

**Supplementary Table 6.** Summary of genes in the neurological manifestation subgroups. Note: 'Normal' refers to the patients diagnosed with BD who do not exhibit neurological manifestation.

| Gene | Neurological manifestation  (total = 2) | Normal  (total = 18) | OR | 95% CI | P |
| --- | --- | --- | --- | --- | --- |
| *TTN* | 1/2 (50%) | 9/16 (56%) | 0.78 | 0.0-69.6 | 1 |
| *FOXO3* | 0/2 (0%) | 7/16 (44%) | 0 | 0.0-8.44 | 0.50 |
| *OR4C5* | 2/2 (100%) | 15/18 (83%) | Inf | 0.0-Inf | 1 |
| *GXYLT1* | 2/2 (100%) | 13/17 (76%) | Inf | 0.0-Inf | 1 |
| *ERN1* | 0/0 (-%) | 2/2 (100%) | not estimable | N/A | N/A |
| *SIPA1L3* | 0/2 (0%) | 5/18 (28%) | 0 | 0.0-16.8 | 1 |

OR, odds ratio; CI, confidence interval; P, p-value of association analysis; Inf, infinite (due to zero counts in one or more cells, reflecting the mathematical boundary conditions of Fisher's exact test); N/A, not applicable (due to absence of comparator group).

**Supplementary Table 7.** Summary of genes in the positive pathergy test subgroups. Note: 'Normal' refers to the patients diagnosed with BD who do not exhibit positive pathergy test.

| Gene | Positive pathergy test  (total = 2) | Normal  (total = 18) | OR | 95% CI | P |
| --- | --- | --- | --- | --- | --- |
| *TTN* | 0/2 (0%) | 10/16 (63%) | 0.0 | 0.0-4.1 | 0.18 |
| *FOXO3* | 1/2 (50%) | 6/16 (38%) | 1.62 | 0.0-143.5 | 1 |
| *OR4C5* | 2/2 (100%) | 15/18 (83%) | Inf. | 0.0-Inf | 1 |
| *GXYLT1* | 2/2 (100%) | 13/17 (76%) | Inf. | 0.0-Inf | 1 |
| *ERN1* | 0/0 (-%) | 2/2 (100%) | not estimable | N/A | N/A |
| *SIPA1L3* | 0/2 (0%) | 5/18 (28%) | 0.0 | 0.0-16.8 | 1 |

OR, odds ratio; CI, confidence interval; P, p-value of association analysis; Inf, infinite (due to zero counts in one or more cells, reflecting the mathematical boundary conditions of Fisher's exact test); N/A, not applicable (due to absence of comparator group).
